# Clay-Polymer Nanocomposites Prepared by Reactive Melt Extrusion for Sustained Drug Release

**DOI:** 10.3390/pharmaceutics12010051

**Published:** 2020-01-07

**Authors:** Xu Liu, Xingyu Lu, Yongchao Su, Eucharist Kun, Feng Zhang

**Affiliations:** 1 College of Pharmacy, the University of Texas at Austin, 2409 University Avenue, A1920, Austin, TX 78712, USA; xliu@utexas.edu (X.L.); yongchao.su@merck.com (Y.S.); eucharistkun@gmail.com (E.K.); 2 Pharmaceutical Sciences, Merck & Co., Inc., Kenilworth, NJ 07033, USA; luxingyu@westlake.edu.cn

**Keywords:** reactive melt extrusion, nanocomposites, polymer–clay complex, controlled drug delivery system, release mechanism

## Abstract

Clay–polymer nanocomposites have exhibited a great potential as carriers for controlled release drug delivery. This study aims to prepare exfoliated montmorillonite–Eudragit RS nanocomposites using reactive melt extrusion and investigate the influence of claying loading, clay types (sodium montmorillonite (Cloisite Na) vs. organomodified montmorillonite (Cloisite 20)) on clay–polymer interactions and drug release properties. The clays were used as the filler material at various levels in Eudragit RS and theophylline was used as the active pharmaceutical ingredient. The resulting structure of the nanocomposites was characterized using TEM (transmission electron microscopy) and XRPD (X-ray powder diffraction). The hygroscopicity of the nanocomposites was investigated using DVS (dynamic vapor sorption). The effect of the interfacial interaction between the polymer and clay sheet, the clay loading as well as the clay type on the drug release behavior were further studied by dissolution testing. TEM and XRPD data show that when the clay content is increased from 5% to 15% by weight, the nanocomposite’s structure switches from a fully exfoliated state to intercalated structures or partial exfoliation with stacked clay layers. FT-IR (fourier transform infrared spectroscopy) and ssNMR (solid-state NMR) results suggest that Cloisite Na and Cloisite 20 layers exhibit different interaction strengths with polymer networks by creating compacted complex structures. The addition of nanoclay in the formulation could robustly adjust drug release profiles, and the clay concentration and type are important factors that affect the crossing-linking density of the nanocomposites by adjusting the drug release properties. This study indicates that the clay–Eudragit RS nanocomposites provide an improved oral controlled drug delivery system that minimizes the drug dosing frequency, potentially leading to improved patient compliance.

## 1. Introduction

Oral controlled drug delivery systems are a recognized protocol to prepare materials that can effectively encapsulate drug molecules and release them at the target site for a defined period of time and in a controlled manner. In addition to improving the drug efficacy, specificity, therapeutic index and tolerability of corresponding drugs, oral controlled drug delivery systems can also reduce the patient expenses as well as the risks of toxicity [1,2]. Because of their multiple and unique advantages, oral controlled drug delivery systems have attracted intense interest from pharmaceutical scientists and formulators for over four decades. Meanwhile, innovative controlled release formulations have remarkably driven the oral drug delivery market to soar in recent years [3,4]. The incorporation of active pharmaceutical ingredients (API) or biological molecules into the biodegradable polymers for a controlled release application has increased dramatically [5].

Hot-melt extrusion (HME) of biodegradable polymers with API, for controlling or modifying the drug release, has received increased attention in the pharmaceutical literature in recent years [6,7]. Compared with other classical preparing techniques, the benefits of HME include being solvent-free, having a high-throughput continuous process, and ease of scaling up [8]. During the HME process, drug, polymer and other excipients are first introduced into the barrel at different temperature settings and feed rates. The rotating screws then mix and melt the materials using heat and an intense mechanical shearing force to achieve distributive and dispersive mixing and excellent homogeneity. The molecular level mixing allows close contact between API and excipients at high frequencies. This makes HME an ideal process for the solid-state chemical reaction. In general, reactive melt extrusion (RME) has referred to combining polymer melt extrusion and chemical reactions into a single extrusion process carried out continuously in an extruder [9]. RME has been widely applied in the plastic and food industries to enhance the properties of materials and products, such as polymer chemical modification and food digestibility [10,11]. Recently, RME has been introduced in pharmaceutical areas to prepare cocrystal [12], coamorphous [13], salt [14], and polyelectrolytes’ complexes [15] to improve the oral bioavailability of poorly water soluble drugs. Although there is an increasing number of HME studies in pharmaceutics, the study of RME for sustained release delivery remains limited. In this study, we utilize RME to prepare clay–polymer nanocomposites for sustained drug release.

Nanocomposites based on polymer and clay are advanced materials for the development of controlled drug delivery systems due to their versatile properties such as (1) high drug encapsulation efficiency, (2) enhanced stability of API against pH variation and enzyme action, (3) low burst release of the drug, and (4) a controlled and targeted drug release profile [16,17,18]. In general, nanocomposites are dispersions of two or more components at the nanometric scale with optimized properties compared to the pure materials, which can be obtained by dispersing clay layers or sheets into a polymeric matrix [19,20]. Clay–polymer nanocomposites can be prepared by various processes, such as melt blending, solution blending, in situ polymerization, and mechano-chemical processing [21,22]. The intercalation of layered structures with polymer chains can be performed by noncovalent bonding involving a cation or anion exchange reaction [23]. Through the interaction between clay and polymer, nanocomposites provide different characteristics from the parent components, including barrier effects, swelling index, mucoadhesion ability and mechanical and thermal stability. The barrier properties of the polymer-based nanocomposites play an important role in determining the drug release profile, dissolution rate, drug uptake and release mechanism [24]. The presence of clays in the polymer matrix strengthens the barrier properties by acting as release retardants for drugs and carriers, thereby promoting stable, controlled drug release in the dissolution media, increasing the solubility of the API, and conferring improved mechanical and thermal properties to the nanocomposites [25,26]. All of these properties are strongly influenced by the surface area and ion exchange capacity of the clay, the type of interactions between clay and polymer and the clay/polymer ratio [27].

There are several different types of clays used in the drug delivery systems, such as kaolin, montmorillonite, saponite, laponite and halloysite [16,28]. Montmorillonite (MMT) has become popular among other clays because of its high availability, environment friendliness, and well-studied chemistry. MMT is a natural material with low or null toxicity, high internal surface area, high cation exchange capacity (CEC), high adsorption and swelling ability, good biocompatibility and, furthermore, it is a material “generally recognized as safe” (GRAS) by the FDA [18,29,30]. MMT is a layered hydrated aluminum silicate which belongs to the smectite group of phyllosilicates. The layer thickness of each platelet is in the range of 1 nm and the lateral dimension is approximately 200 nm. Cloisite Na is a natural MMT without any modification and Cloisite 20 is an organic modified MMT with quaternary ammonium salts. Because of the appearance of long alkyl chains, the interlayer spacing of MMT is enhanced, resulting in hydrophobic MMT. MMT has the empirical formula Al_2_O_3_·4SiO_2_·H_2_O, and due to the substitution of some Al^3+^ with Mg^2+^, it carries an overall negative charge on its hydrophilic platelet surface. Because of its permanent negative charge, MMT has been used to prepare nanocomposites by electrostatic interactions with cationic polymers such as chitosan and gelatin [29,31]. In this study, layered silicate nanocomposites have been prepared by melt intercalation. 

There are many studies which focus on the application of nanocomposites as drug delivery systems [32,33]. However, there are few papers focusing on the influence of the clay–polymer interaction on the drug release profiles [34]. No study has compared the effect of Cloisite Na and Cloisite 20 on the resulting Cloisite/Eudragit RS nanocomposites so far. There are also few researches on their structural confinement properties and on the mechanisms that underlie their interactions with polymer. In 2017, Bee et al. investigated the effect of Cloisite Na and Cloisite 20 on the morphology, mechanical and thermal properties of the resulting poly (methyl methacrylate) (PMMA) nanocomposites prepared using a Brabender mixer [35]. They found that Cloisite 20 shows better compatibility with PMMA compared with Cloisite Na. It was found that Cloisite 20 formed nanocomposites with PMMA, while Cloisite Na only formed microcomposites. The results show that the properties of the Cloisite 20 nanocomposites exceeded the neat PMMA and PMMA/Cloisite Na microcomposites, which is attributed to the formation of more a favorable polymer–filler interaction. However, the authors overlooked the influence of the composites’ structure on the polymer–filler interaction since an insufficient mixing process might limit the polymer–filler interaction. Considering the importance of the water permeation and the different surface properties of Cloisite Na and Cloisite 20, our hypothesis in this study was that the dispersion of Cloisite nanoplatelets into the Eudragit RS matrix might easily adjust the water uptake and the drug release rate and the variation of the interactions between polymers and pristine clay or organically modified clay and polymers would impact the drug release behaviors.

**Figure 1:**
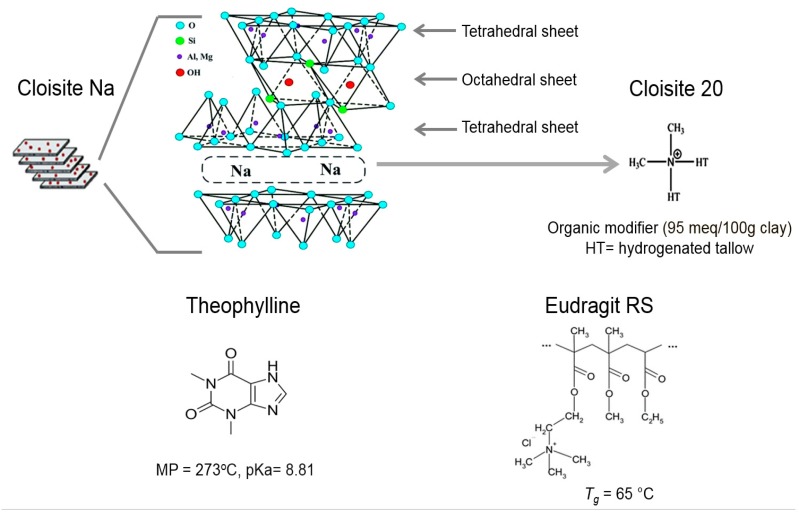
Chemical structures and critical properties of Cloisite Na, Cloisite 20, theophylline and Eudragit RS.

In this study, Cloisite Na and Cloisite 20 were selected as model clays. The chemical formula of Cloisite Na is Al_2_O_3_·4SiO_2_·H_2_O, the basal spacing is 1.2 nm and its cation exchange capacity has been reported to be approximately 92 meq/100 g [30]. Cloisite 20 is prepared from Cloisite Na with hydrogenated tallow (HT, ~65% C18; ~30% C16; and ~5% C14) and the modifier concentration is 95 meq/100 g and the basal spacing is 2.4 nm. Eudragit RS, a cationic copolymer of ethyl acrylate, methyl methacrylate, and a low content of methacrylic acid ester with quaternary ammonium groups, was selected as the polymer carrier. Figure 1 presents the chemical structures and critical attributes of the drug and excipients used in this study. The objective of this study was to compare the difference of the interfacial interactions between polymers and pristine clay and organically modified clay in sustained release hydrophobic matrices prepared by hot melt extrusion, and to investigate the influence of the clay–polymer interactions on the physicochemical properties of the extrudates. The nanocomposite structure and possible interaction between clays and polymer were investigated by DSC, XRPD, TEM, FT-IR, ssNMR and DVS. Dissolution testing was performed in pH 6.8 phosphate buffer dissolution medium to investigate the influence of the clay loading, clay type and polymer–clay interaction on permeability and drug release properties.

## 2. Materials and Methods

### 2.1. Materials

Cloisite Na and Cloisite 20 were donated by Southern Clay Products, Inc (Gonzales, TX, USA). Eudragit^®^ RS was donated by Evonik Industries (Darmstadt, Germany). Anhydrous theophylline USP was purchased from Acros Organics (Pittsburgh, PA, USA). Sodium phosphate monobasic and sodium phosphate dibasic were purchased from Fisher Scientific (Waltham, MA, USA). All other reagents and solvents were analysis grade or better.

### 2.2. Methods

#### 2.2.1. Preparation of Nanocomposites

**Figure 2:**
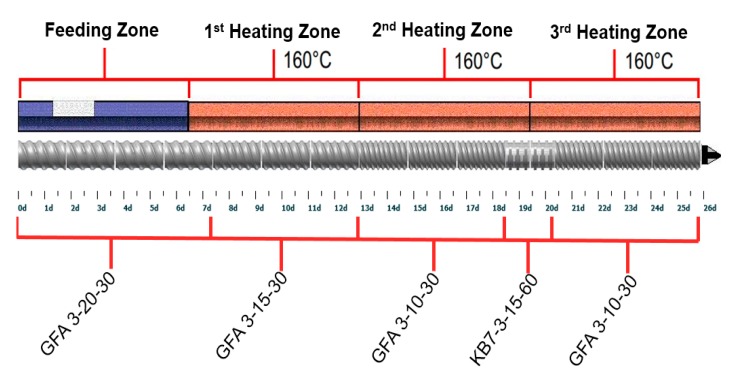
Screw configuration and barrel temperature for reactive melt extrusion of theophylline granules.

Clay–polymer nanocomposites were prepared using a Leistritz Nano 16 extruder (Leistritz Corporation, Allendale, NJ, USA). The composition of the various powder blends is summarized in Table 1. A Turbula^®^ Shaker-Mixer (Glen Mills, Clifton, NJ, USA) was used to prepare powder blends for extrusion. A twin-screw volumetric feeder (Brabender Technologie, ON, Canada) was used to feed the powder blends at a rate of 300 g/h. The screw profile is shown in Figure 2. The screw speed was set at 100 rpm and the barrel temperature was at 160 °C. 

Preparation of theophylline granules was performed in two steps. In the first step, clay and polymer nanocomposites were prepared. In the second step, blends of the milled nanocomposites and theophylline (20% loading) were extruded to incorporate the drug. Milling of extrudates was carried out using a Comill (Quadro Waterloo, ON, Canada). The milled extrudates (theophylline granules) were stored in a desiccated chamber at room temperature for further analysis.

Data in the screw code (GFA X-XX-XX) represent the trilobal screw, pitch length (mm) and screw length (mm), respectively. Data in the screw code (KB X-X-XX-XX) indicate the number of kneading segments, trilobal screw, screw length (mm) and the angle (°).

#### 2.2.2. Transmission Electron (TEM) and Scanning Electronic Microscopy (SEM)

Dispersions of Cloisite Na and Cloisite 20 in Eudragit RS were examined using a high-resolution FEI Tecnai TEM (ThermoFisher Scientific, Hillsboro, OR, USA) with an acceleration voltage of 100 kV. The exposure time varied from 0 to 100 s. Ultrathin sections of nanocomposites were prepared with a Leica Ultracut UC7 ultramicrotome (Leica Microsystems Inc., Buffalo Grove, IL, USA) equipped with a diamond knife. All samples were placed on 200 mesh copper grids before loading into the instrument. The surface morphology of the theophylline granules following dissolution testing was examined using Zeiss Supra40 SEM (Carl Zeiss, Thornwood, NY, USA). All samples were tested with 5 kV accelerating voltage and 30 µm aperture coated with 15 nm Pt. 

#### 2.2.3. Differential Scanning Calorimetry (DSC)

DSC analysis was performed using a Model Q-2000 DSC (TA Instruments, Newcastle, DE, USA) equipped with the RCS 90 (TA Instrument, Newcastle, DE, USA) refrigerated cooling system accessory under a dry nitrogen purge (50 mL/min). Calibration was carried out with an indium standard and an empty TA aluminum pan was used as the reference. Samples were accurately measured (3–5 mg) in aluminum pans and crimped with aluminum lids. Samples were heated from 20 to 350 °C at a heating rate of 10 °C/min. The DSC data were analyzed using the TA Universal Analysis 2000 software (TA Instrument, Newcastle, DE, USA).

#### 2.2.4. X-ray Powder Diffraction (XRPD)

XRPD was performed using a Rigaku MiniFlex 600 X-ray diffractometer (Rigaku Corporation, Tokyo, Japan) equipped with a copper X-ray tube (wavelength λ = 0.154 nm). Milled samples were placed on silicon sample holders and the measurements were performed with an acceleration voltage of 40 kV, and a current of 15 mA, 2-theta angles between 5° and 45° with a scan speed of 1°/min, and a step size of 0.02°. The results were analyzed with the MDI Jade 8.5 software (Material Data, Inc., Livermore, CA, USA) and plotted with OriginLab version 9.0 software (OriginLab Corporation, Northampton, MA, USA). The thickness of the silicate layer was calculated using Bragg’s equation:

nλ=2dsinθ

where *n* is order reflection; *λ* is the X-ray wavelength (1.54 A); *θ* is the angle of the basal spacing peak of clay; and *d* is the thickness of the clay silicate layer.

#### 2.2.5. Fourier Transform Infrared Spectroscopy (FT-IR)

Molecular interactions between clay and polymer chain were measured with FT-IR. The analyses were performed using a Thermo Nicolet iS50 spectrometer (Waltham, MA, USA). Samples were placed on a germanium crystal surface. A fixed torque was applied using the built-in pressure tower to reach uniform contact between the sample and the crystal. All measurements were carried out at ambient room temperature with a total of 32 scans at a 4 cm^−1^ resolution from 600 cm^−1^ to 4000 cm^−1^. The peak positions were analyzed using the OMNIC software peak picking function (ThermoFisher Scientific, Waltham, MA, USA). 

#### 2.2.6. Solid-State NMR (ssNMR)

All ssNMR experiments were examined on a triple-channel 400 MHz Bruker AVANCE III spectrometer (Bruker BioSpin, Billerica, MA, USA) in the Biopharmaceutical NMR Lab (BNL) at Pharmaceutical Sciences, MRL (Merck & Co., Inc. West Point, PA, USA). One-dimensional (1D) ^13^C spectra were obtained at magic angle spinning (MAS) of 12 kHz with a Bruker 4 mm HFX MAS probe in double-resonance mode tuned to ^1^H and ^13^C-nucleus frequencies. ^13^C spectra were referenced to the tetramethylsilane (TMS). All spectra were obtained at 298 K and processed in Bruker Topspin software (Bruker Corporation, Billerica, MA, USA). 1D ^13^C cross-polarization (CP) transfers were performed with a radio-frequency (RF) strength of 80–100 kHz during a 2 ms contact time. The power level was ramped linearly over a depth of 15–20 kHz on the ^1^H channel to enhance CP efficiency. ^1^H heteronuclear decoupling for ^13^C was performed at an RF strength of 100 kHz using the SPINAL-64 pulse sequence. ^1^H spin-lattice relaxation times in the laboratory frame (T_1_) were determined by ^13^C-detected saturation recovery experiments [36].

#### 2.2.7. Dynamic Vapor Sorption (DVS)

The water sorption-desorption isotherms of the clay dispersion were determined using a TA VTI-SA+DVS analyzer (TA Instruments, Newcastle, DE, USA). Two relative humidity cycles were performed for each sample at 25 °C and water was used as the testing medium. In each cycle, relative humidity was raised in 5 steps, 15% ramp per step, from 0% to 75% and then back to 0%. A rate of change in mass per time unit (dm/dt) of 0.001%/min was set as the equilibration parameter. At each stage, DVS held the set parameters for 30 min once dm/dt reached 0.001%/min. The DVS water sorption limits were calculated by averaging the mass difference between 75% relative humidity and 0% relative humidity of each cycle.

#### 2.2.8. Dissolution Testing

Dissolution testing of theophylline granules with diameter range of 500–595 µm was performed in 900 mL of phosphate buffer with a pH of 6.8 at 37 °C ± 0.5 °C using the USP Type II apparatus (Varian VK7025, Agilent Technology Inc., Santa Clara, CA) at a paddle speed of 75 RPM. In total, 500 mg of theophylline granules were introduced into each dissolution vessel. Three milliliter dissolution samples were withdrawn at specific time points (0.5, 1, 2, 4, 6, 9, 12, and 24 h) using an autosampler (Varian VK7025, Agilent Technology Inc., Santa Clara, CA, USA) without sample replacement. The samples were filtered through Vankel Full Flow 10 µm filters. The drug concentration was analyzed with a UV-Vis spectrophotometer at 245 nm (Infinite M200, Tecan Group Ltd., Mannedorf, Switzerland). All samples were tested in triplicate. 

## 3. Results and Discussion

### 3.1. Preparation of Cloisite–Eudragit RS Nanocomposites and Theophylline Granules

Four methods commonly used to prepare clay–polymer nanocomposites are in situ template synthesis, solution intercalation, in situ intercalative polymerization, and melt intercalation. In this study, melt intercalation was applied to prepare clay–Eudragit RS nanocomposites. Melt intercalation was carried out using an intermeshing corotating twin-screw extruder. Cloisite–Eudragit RS nanocomposites were initially prepared using melt extrusion. Powder blends of the milled nanocomposites and theophylline were then extruded to prepare theophylline granules. The composition, extrusion torque, and extrudate images are listed in Table 1. The advantage of nanocomposites is that the relatively small amount of clay loading (1%–20% by weight) results in the best combination of property improvements to the hybrid materials [37]. Due to the torque limitation of the twin-screw extruder in this study, the maximum clay loading was set at 15%. At a given clay loading, Cloisite Na-containing formulations demonstrated higher extrusion torque than Cloisite 20-containing formulations, indicating stronger Cloisite Na–Eudragit RS interactions.

As shown in Table 1, transparent Eudragit RS extrudate became translucent with the incorporation of Cloisite. Theophylline granules were opaque, indicating that the drug was not fully solubilized in the extrudates. Both the dispersion of Cloisite in Eudragit RS and crystallinity of theophylline were thoroughly characterized, and the results are presented in later sections. 

### 3.2. Characterization of Cloisite–Eudragit RS Nanocomposites

#### 3.2.1. The Nanocomposites’ Structure

Physical properties such as mechanical strength and permeability are defined by the micro-structure of the clay–polymer nanocomposite. The preparation of nanocomposites requires uniform dispersion of the layered silicate in the polymer matrix at the nanometer scale. Based on the physical state of the clay layers and their distribution state, clay–polymer nanocomposites can be categorized into three types: aggregated, intercalated, and exfoliated [38]. In the aggregated structure, the clay tactoids are well dispersed in the polymer matrix, but the single clay layers are not delaminated. In the intercalated structure, the clay tactoids are partially delaminated and the polymer chains diffuse into the galleries between them. In the exfolicated structure, the clay tactoids are completely broken apart into single layered platelets, which are homogeneously distributed in the polymer matrix. When aggregation of the clay platelets presents due to intercalation without complete exfolication, then the tortuous path is correspondingly reduced. As a result, the exfoliated structure is the most ideal state as it can provide excellent barrier and mechanical properties at low clay contents [21,26]. In general, the purpose of compounding clay–polymer nanocomposites is to achieve complete exfoliation of the layered silicate in a polymer matrix. During the reactive melt extrusion process, delamination and dispersion of the clay particles might occur in two steps: (1) the clay particles shear apart and the polymer chains intercalate to clay galleries and (2) polymer chains enter the galleries of the clay and push platelets apart, which eventually allows the platelets to peel off the intercalated clay stack [38]. 

**Figure 3:**
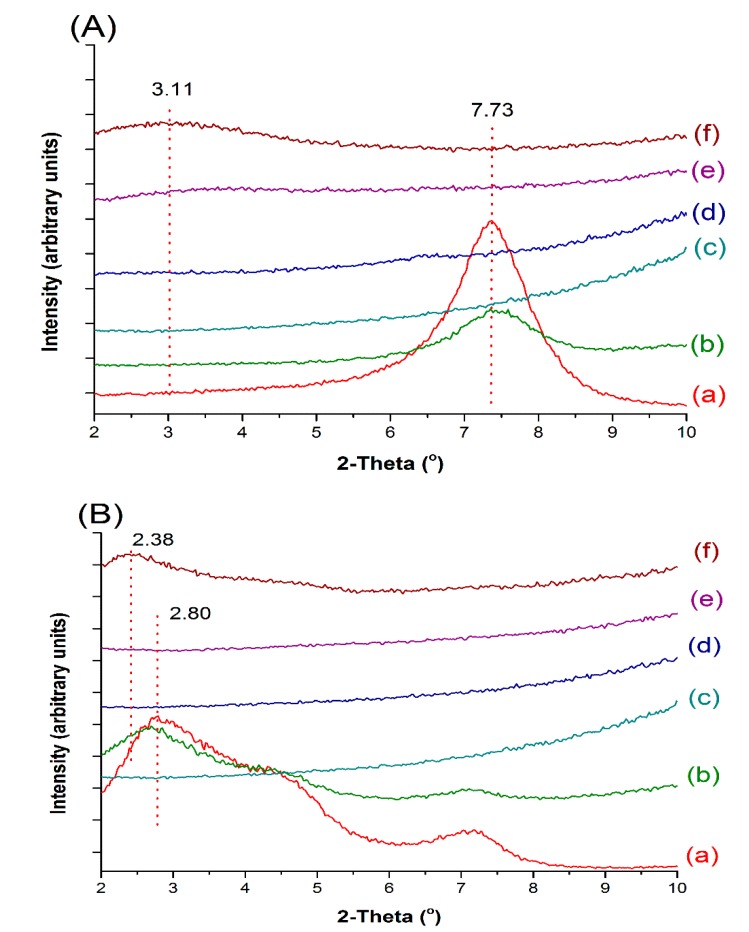
XRPD patterns of Cloisite, Eudragit RS, and their extruded nanocomposites. (**A**): (a) Cloisite Na; (b) 5% Cloisite Na–95% Eudragit RS physical mixture; (c) Eudragit RS; (d) nanocomposite containing 5% Cloisite Na; (e) nanocomposite containing 10% Cloisite Na; (f) nanocomposite containing 15% Cloisite Na. (**B**): (a) Cloisite 20; (b) 5% Cloisite 20%–95% Eudragit RS physical mixture; (c) Eudragit RS; (d) nanocomposite containing 5% Cloisite Na; (e) nanocomposite containing 10% Cloisite Na; (f) nanocomposite containing 15% Cloisite Na.

The structure of Cloisite Na–Eudragit RS nanocomposites was investigated using XRPD and TEM techniques. As shown in Figure 3A, the major diffraction peak of Cloisite Na at 7.73° (Figure 3A(a)), corresponding to a mean interlayer spacing of 11.8 nm, was present in the diffraction pattern (Figure 3A(b)) of the Cloisite Na–Eudragit RS physical mixture. At 5% and 10% Cloisite Na loadings, the peak at 7.73° was absent in the XRPD patterns of the nanocomposites (Figure 3A(d–f)), indicating complete exfoliation of nanoclay platelets in the polymer matrix. At 15% Cloisite Na loading, a new broad peak at 3.11° implied the formation of an ordered intercalated nanocomposite. The decrease in 2θ angle reflects the enlarged d-spacing of clay platelets and increased gallery gap due to the intercalation of Eudragit RS. 

Similar XRPD patterns were also observed with Cloisite 20-Eudragit RS nanocomposites. Cloisite 20 exhibited a diffraction peak at 2.80°, corresponding to a d-spacing of 2.42 nm (Figure 3B). Positioning of the diffraction peak at such a low 2θ angle is due to the intercalation by the tallow surfactants. At 5% and 10% clay loading, Cloisite 20 was fully exfoliated in the polymer matrix. At 15% clay loading, a broad peak at 2θ of 2.38° corresponding to a gallery gap of 2.42 nm was observed, due to the intercalation of the polymer chain. 

**Figure 4:**
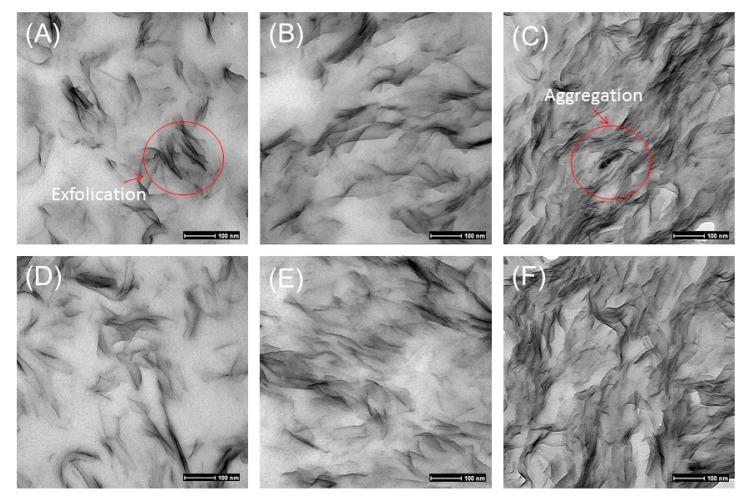
TEM images of Cloisite–Eudragit RS nanocomposites prepared using RME. (**A**) 5% Cloisite Na, (**B**) 10% Cloisite Na, (**C**) 15% Cloisite Na, (**D**) 5% Cloisite 20, (**E**) 10% Cloisite 20, (**F**) 15% Cloisite 20.

TEM results agreed well with XRPD results. Dark lines or areas in the TEM images represent clay, and the off-white phase was Eudragit RS (Figure 4). The TEM images of Cloisite–Eudragit RS showed the exfoliated or intercalated structure, depending on clay loading. Fine and uniform dispersion of Cloisite sheets in Eudragit RS was observed. Most Cloisite sheets aligned perpendicularly to the sample cutting surface. At 5% and 10% loadings, Cloisite Na and Cloisite 20 were uniformly dispersed in the Eudragit RS matrix and an exfoliated nanocomposite structure was observed. At 15% clay loading, the clay sheets became denser and the intercalated nanocomposite structures were observed. It was concluded that the clay type did not impact the dispersion status of the clay in Eudragit RS. The coherent order of the stacked layers strongly depended on the clay loading. 

Exfoliation of Cloisite clays by Eudragit RS was mainly driven by the ionic interactions. As shown in Figure 1, quaternary ammonium group in Eudragit RS is positively charged while the clay sheet surface is negatively charged. Since the drug molecules could not penetrate through the clay sheets, the increase in tortuosity as a result of ionic interaction would lead to slower diffusion [39]. The interaction could also reduce the hygroscopicity of Eudragit RS because less quaternary ammonium groups are available to interact with water molecules following the clay–polymer complexation. 

At 5% and 10% clay loadings, all nanocomposites showed similar exfoliated structures regardless of clay types. For Cloisite 20, 86% of the intercalating sites are blocked by the ternary ammonium surfactant. The decrease in the available intercalating sites of Cloisite 20 could lead to fewer interactions between the clay and Eudragit RS, potentially impacting drug release behaviors. FT-IR and ssNMR techniques were applied to investigate the molecular mechanisms of Eudragit RS–Cloisite interactions. 

#### 3.2.2. Investigation of Cloisite–Eudragit RS Interactions Using FT-IR

Due to isomorphous substitution, the silicate layers of Cloisite are negatively charged, which is balanced by interlayer Na^+^. During the extrusion process, the ion exchange reaction took place, and the quaternary ammonium groups of Eudragit RS replace the Na^+^ and get ionically bound to the silicate layers. This type of interaction has been reported in other nanocomposites prepared with MMT and cationic polymers such as chitosan and gelatin [29,31,40].

**Figure 5:**
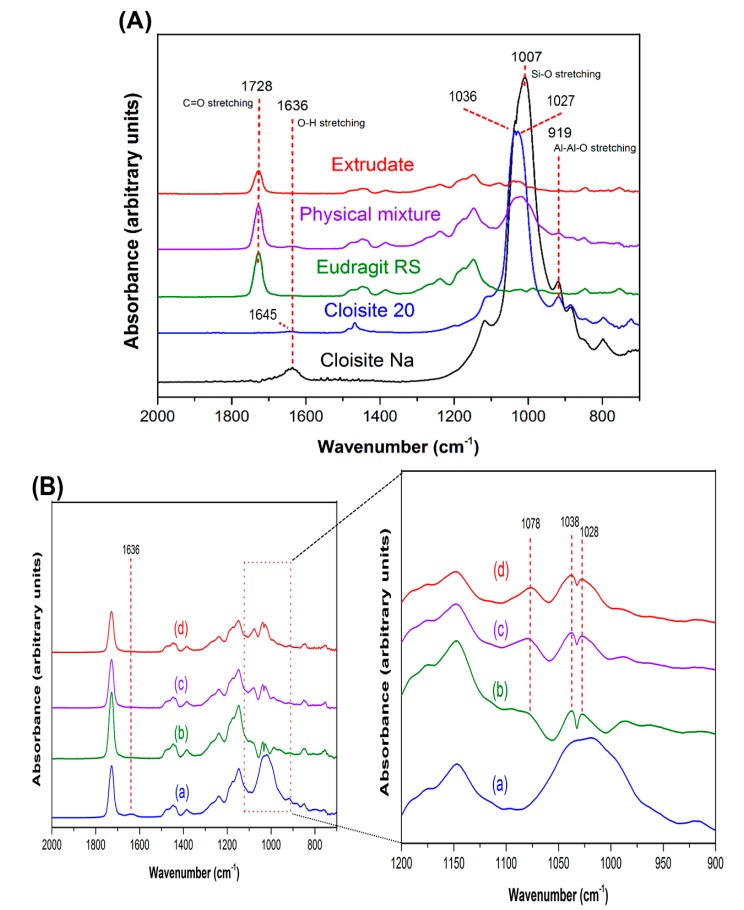
FT-IR spectra of extruded Cloisite–Eudragit RS nanocomposites. (**A**) 10% Cloisite Na–90% Eudragit RS nanocomposite, individual components, and their physical mixture; (**B**): (a) 5% Cloisite Na–95% Eudragit RS physical mixture; (b) Cloisite Na nanocomposite at 5% clay loading; (c) Cloisite Na nanocomposite at 10% clay loading; (d) Cloisite Na nanocomposite at 15% clay loading.

Interactions between Cloisite and Eudragit RS were studied using FT-IR technique. The functional groups involved in molecular interaction could be reflected in the emergence of a new band, shift in band position, or change in band shape in FT-IR spectra [41]. The band assignment for Cloisite Na, Cloisite 20 and Eudragit RS FT-IR spectra (Figure 5A) is summarized in Table 2. The bands indicative of the Cloisite–Eudragit RS interactions are in the range of 500–1800 cm^−1^ [42]. These bands are associated with bending, deformation, and stretching of Si-O-Si, structural OH, and adsorbed water. 

In the IR spectrum of Cloisite Na, the band at 1636 cm^−1^ (Figure 5A) is attributed to in-plane bending of water in the hydration sphere of the interlayer Na^+^ ion [42]. The broad band in the region of 950–1100 cm^−1^ is associated with Si-O stretching vibrations [43]. For Cloisite 20, the ionic interactions between the intercalated surfactant and silicate surfaces significantly impact the arrangement of SiO_4_ tetrahedral layers. The loading and alkyl chain length of the surfactant significantly impact the shape and wavenumber of the bands discussed above. In the FT-IR spectrum of Cloisite 20, the frequency of in-plane bending of water in the hydration sphere of the interlayer Na^+^ ion shifted from 1636 cm^−1^ to 1645 cm^−1^, while the intensity of this band decreased significantly. In addition, the peak of Si-O stretching vibrations in plane split into two peaks, one at 1036 cm^−1^ and another at 1027 cm^−1^ (Figure 5A). 

Two notable changes in the FT-IR spectra of the clay–polymer nanocomposites indicate the interactions between Cloisite Na and Eudragit RS (Figure 5B). Firstly, the 1636 cm^−1^ band indicative of water molecules hydrating the interlayer Na^+^ disappeared because of the displacement of the interlayer Na^+^ and associated water molecules by Eudragit RS [44,45]. Secondly, the board band in the region of 950–1100 cm^−1^, corresponding to the vibration of Si-O, split into two peaks at 1038 and 1028 cm^−1^. The splitting of the Si-O vibration band is affected by not only the chemical nature of the intercalated components, but also the basal spacing of the clay sheets. Because smaller basal spacing leads to less significant splitting, the splitting was less at higher Cloisite Na loadings (by comparing Figure 5B), As illustrated in the XRPD and TEM results discussed earlier, the interlayer space decreases significantly with increased the clay loading. In addition to peak splitting, a new band attributed to perpendicular Si-O stretching was observed at 1078 cm^−1^ [46]. The intercalation of the Eudragit RS or surfactants into the Cloisite interlayer space resulted in a marked interlayer swelling during which a perpendicular adsorbate orientation is reached, accounting for the appearance of the new peak at 1078 cm^−1^. Furthermore, with the decrease in clay loading, SiO_4_ tetrahedra oriented toward a more ordered arrangement. Therefore, perpendicular Si-O vibration became more significant [46,47]. The similar results were observed in Cloisite 20–Eudragit RS nanocomposites (supplemental data, Figure S1). It is difficult to differentiate the interaction between Eudragit RS and Cloisite Na or Cloisite 20 at the same clay loading level through FT-IR analysis. 

The results not only indicate the clays were entrapped in the polymer matrix, but also that the clay layers interacted with polymer network to create a compacted complex structure for both Cloisite Na and Cloisite 20.

#### 3.2.3. Investigation of Cloisite Na–Eudragit RS Interactions Using ssNMR

**Figure 6:**
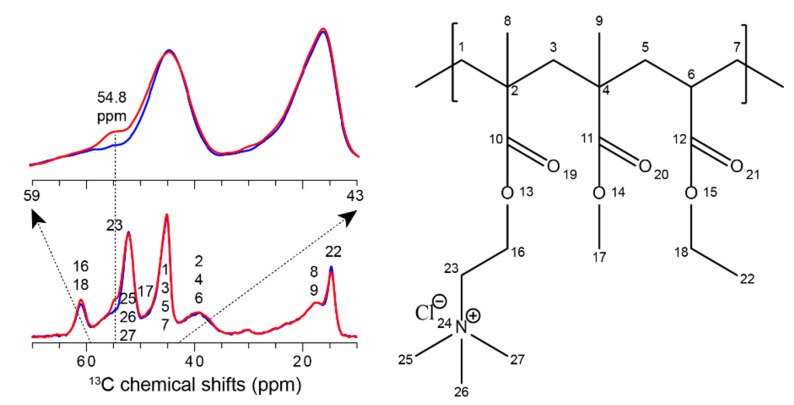
1D 13C spectral comparison between Eudragit RS (red) and Cloisite Na–Eudragit RS dispersions (blue). Enlarged spectra were displayed in an overlaid manner. Tentative 13C chemical shift assignments are labeled using 13C numbers correspondingly shown in the Eudragit RS molecule structure.

In previous studies, ssNMR was utilized to characterize the molecular interaction between Cloisite–polymer nanocomposites. For example, the dynamic behavior of the local domains in between Cloisite and polymer is considered of important to understand the macroscopic properties of a nanocomposite [48]. Molecular motions at frequencies of the order of the Larmor frequency (MHz regime) can strongly influence the nuclear spin-lattice relaxation processes in the laboratory (T_1_). We utilized ^13^C-detected saturation recovery experiments to measure the Eudragit RS ^1^H spin-lattice relaxation times in the laboratory frame [36]. The ^1^H T_1_ of Eudragit RS only was measured as 1.1 s and decreased to 0.8 s upon the incorporation of Cloisite Na, presumably exhibiting enhanced molecular dynamics. As a hypothesis, it may indicate that Eudragit RS molecules were well dispersed between Cloisite Na layers and formed as a flexible dispersion, comparing to its original dense polymer assemblies. Besides molecular motions, ssNMR has been often utilized to probe intermolecular drug–polymer interactions [49]. Therefore, we further analyzed the Cloisite–polymer dispersion using ^13^C CPMAS. The ^13^C resonances of Eudragit RS are tentatively assigned. In Figure 6, The 1D ^13^C spectra comparison exhibits an interesting spectral difference at 54.8 ppm between Eudragit RS only and Cloisite Na–Eudragit RS dispersions. This peak can be tentatively assigned as the polymer C23, adjacent to -N^+^(CH_3_)_3_ in one of sidechains. While relative intensity of all other carbons remains unchanged, the loss of C23 intensity may suggest the perturbation of its surrounding proton network as well as molecular mobility, both of which attenuate the magnetization transfer during ^1^H-to-^13^C CP. For example, C23 can reside in a more diluted proton environment and exhibit faster molecular dynamics if the -N^+^(CH_3_)_3_ sidechain is involved in between Cloisite Na layers. These molecular details will be further investigated by utilizing more quantitative multiCP and two-dimensional site-specific ssNMR experiments in future studies [49,50].

#### 3.2.4. Hygroscopicity of Nanocomposites

Eudragit RS is a copolymer of ethyl acrylate, methyl acrylate, and 3.3% (molar) of methacrylic acid ester with quaternary ammonium groups (trimethylammonioethyl methacrylate chloride). The ammonium groups are present as salt and make the polymer hygroscopic and permeable. Ion exchange interactions between clay and polymer lower the hygroscopicity of Eudragit RS. With the dispersion of silicate layers throughout the polymer matrix, the water barrier properties of the Eudragit RS are expected to be enhanced since water molecules must bypass impenetrable silicate platelets and permeate through a more tortuous diffusion path [37,51]. 

**Figure 7:**
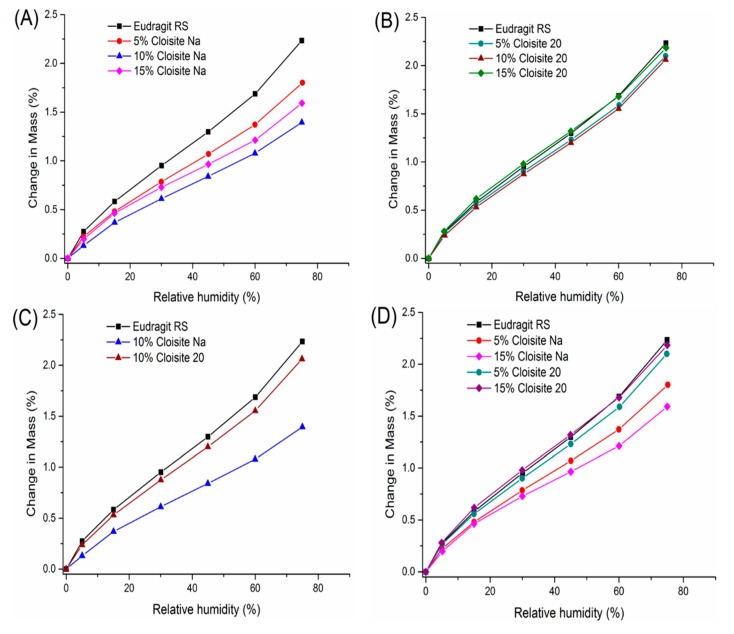
DVS profiles comparison of extruded Cloisite–Eudragit RS nanocomposites (35–50 mesh). (**A**) nanocomposites with different Cloisite Na loadings (**B**) nanocomposites with different Cloisite 20 loadings (**C**) nanocomposites with different clays at 10% loading (**D**) nanocomposites with different clays at 5% and 15% loadings.

The moisture sorption isotherms were determined for Eudragit RS and its Cloisite nanocomposites. As shown in Figure 7A, hygroscopicity of nanocomposites ranked in the following order: 10% Cloisite Na ˂ 15% Cloisite Na ˂ 5% Cloisite Na ˂ Eudragit RS only. The Cloisite Na nanocomposite at 10% clay loading was the least hygroscopic. An initial decrease followed with increase in hygroscopicity beyond a threshold value was also reported with other clay–polymer nanocomposites [51,52,53]. In 2013, Duan et al. reported the lowest water vapor transmission rate at 5% clay loading for MMT-PLA nanocomposites containing 1% to 6% clay. The experimental data agreed well with the predictions from the Nielsen “tortuous path” model [54]. Increase in hygroscopicity above a clay-loading threshold was attributed to the nanoclay agglomeration effect [37]. The increase in water permeability can also be explained by the increasing level of nonexfoliated silicate layers that formed tactoids and intercalated structures [55]. Aggregates in the intercalated structure facilitated the diffusion of water molecules via the connecting pathways along the polymer–clay interfacial zones [56]. In Cloisite–Eudragit RS nanocomposites, Cloisite Na platelets started to aggregate at 15% clay loading due to the intercalation without complete exfoliation. 

In our study, we found that the exfoliated nanocomposites could be achieved at 10% Cloisite Na loading, which shows the maximum water-barrier effect. The exfoliated nanocomposites with higher clay loading could be made probably related with the higher shear stresses generated during the twin screw extrusion process and the good miscibility of Cloisite Na and Eudragit RS.

Yet, the moisture sorption of Cloisite 20–Eudragit RS nanocomposites was similar across different Cloisite 20 loadings (Figure 7B). The equilibrium moisture content of nanocomposites containing 5% and 10% Cloisite 20 was 5.8%, and 7.6% equilibrium moisture content was achieved at 25 °C/75% RH. The 15% Cloisite 20 nanocomposites even show slightly higher water hygroscopicity than Eudragit RS.

At a given clay loading, Cloisite Na is more effective than Cloisite 20 in reducing hygroscopicity of Eudragit RS, even though Cloisite 20 itself is less hygroscopic than Cloisite Na [57,58]. Our DVS data shown the hygroscopicity of Cloisite Na was about 15 times than Cloisite 20 at 25 °C and 75% RH condition (supplemental data, Figure S2). However, Cloisite Na nanocomposites show 32% less hygroscopicity than Cloisite 20 nanocomposites at 25 °C and 75% RH at 10% clay loading (Figure 7C). This is attributed to the stronger clay–Eudragit RS interaction for Cloisite Na. For Cloisite 20–Eudragit RS nanocomposites, the intercalation of the polymer chain to the silicate layers enhances the exfoliation because the surfactant molecules cannot be squeezed out upon collapse of the layer. 

Moisture absorption of Eudragit RS is mainly controlled by its tertiary amine group. Due to the surfactant coating, Cloisite 20 has a lower cation exchange capacity compared with Cloisite Na. As a result, the quaternary ammonium groups of Eudragit RS have stronger interaction with the Cloisite Na. 

At the same clay loading, the transmission rate of water through the composites is more influenced by polymer–silicate layer interactions. The nanofiller did not influence the water sorption capacity in the amorphous domains, however, the polymer interacted with the clay in the interlayer space, leading to the lowered hygroscopicity of this phase. Different types of interactions between the polymer and inorganic platelets may impact the free volume in the matrix, the interfacial regions between the two different phases and the degree of delamination of the silicate layers. A number of studies compare the efficacy of different filler types in specific polymer systems. In 2009, Alexandre et al. also compared the water barrier properties of polyamide 12/organically modified MMT nanocomposites [56]. They found that the water permeability and diffusivity decreased with increasing clay volume fraction up to 2.5%. However, the water barrier effect was not improved by further addition of clay. The loss of barrier properties was attributed to several concomitant effects, such as the change of the polymer crystallinity, the water-induced plasticization and the structure heterogeneity. 

In summary, Cloisite Na nanocomposites are more effective in inhibiting water absorption than Cloisite 20 nanocomposites and this is attributed to the difference in the interactions between Eudragit RS and silicate layers.

### 3.3. Characterization of Cloisite–Eudragit RS Nanocomposites Loaded with Theophylline

#### 3.3.1. Characterization of Physical State of Theophylline in Extrudate

**Figure 8:**
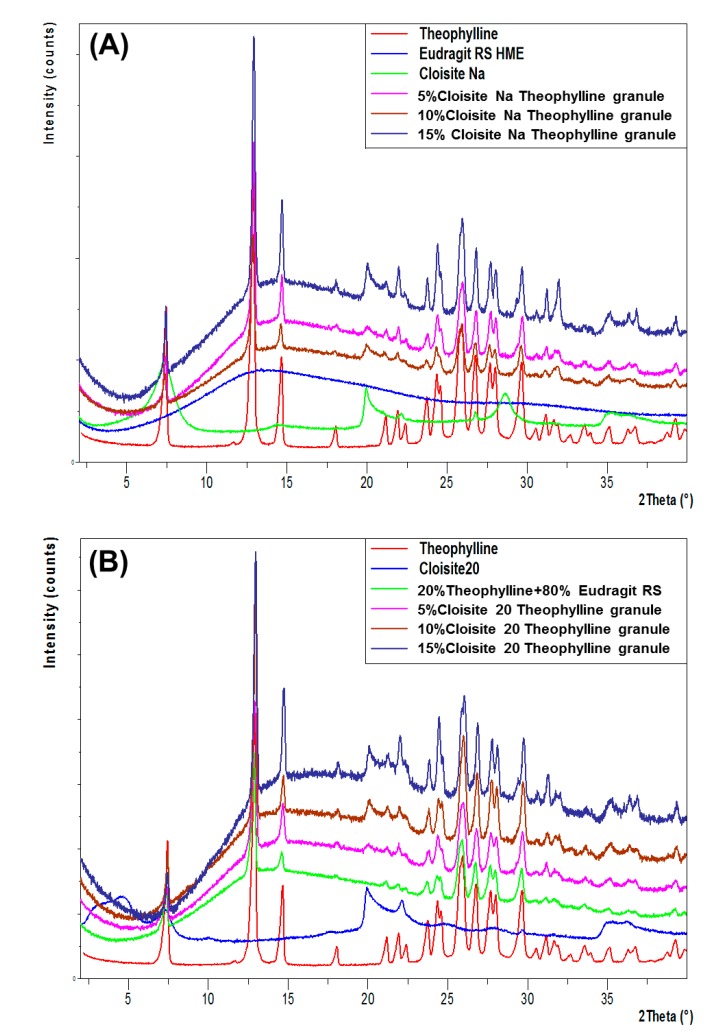
XRPD patterns of theophylline granules (20%) based on Cloisite–Eudragit RS nanocomposites and individual components. (**A**) Cloisite Na–Eudragit RS nanocomposites at different clay to polymer ratios, (**B**) Cloisite 20-Eudragit RS nanocomposites at different clay to polymer ratios.

**Figure 9:**
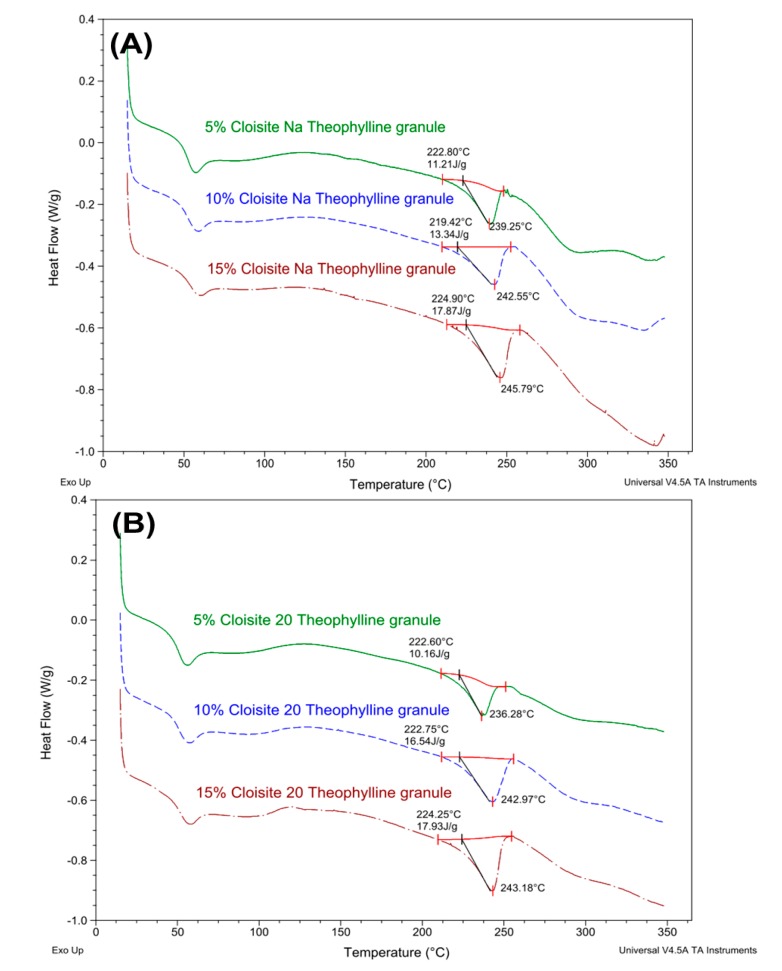
DSC profiles of theophylline granules (20%) based on Cloisite–Eudragit RS nanocomposites. (**A**) Cloisite Na–Eudragit RS nanocomposites at different clay to polymer ratios (**B**) Cloisite 20-Eudragit RS nanocomposites at different clay to polymer ratios.

The theophylline extrudates were prepared at 160 °C, significantly below the melting point (273 °C) of theophylline. As shown in Figure 8, all major characteristic peaks (7.30°, 12.77°, 14.51°, and 25.76°) of theophylline were present in XRPD patterns of Cloisite Na or Cloisite 20-based theophylline granules. The intensity of theophylline diffraction increased with the increase in clay loading. These results indicated that the theophylline was not fully dissolved in the extrudates and part of the drug still exist as crystalline state in the nanocomposite matrix. The DSC data (Figure 9) confirmed the XRPD results. Theophylline melts at 271 °C with a melting enthalpy of 197 J/g (Figure 9A). The higher the clay loading is, the lower the theophylline melting enthalpy of the extrudates. Via DSC analysis, the percentage of theophylline remaining crystalline in Cloisite Na-based formulations was determined to be 45.4%, 54.83%, and 72.43% at 4%, 8%, and 12% clay loading, respectively. Similar results have been observed in Cloisite 20-based formulations (Figure 9B). It is noteworthy that under the same clay loading, the drug crystallinity in Cloisite Na and Cloisite 20-based formulations are similar.

Higher crystalline theophylline content at higher clay loading is due to two factors. Firstly, there is less Eudragit RS to solubilize theophylline in clay–polymer nanocomposites containing with a higher level of clay loading. Secondly, higher clay loading results in less distributive mixing. As discussed earlier, higher clay loading resulted in higher extrusion torque, which was indicative of a higher melt viscosity for the formulation. The distributive mixing during the extrusion process is limited due to the higher viscosity of the extrudates, which result in higher residual crystallinity in the formulation.

In summary, theophylline exists in a partially crystalline state in both Cloisite Na and Cloisite 20 nanocomposite matrices and the clay type does not impact the physical state of drug during the extrusion process.

#### 3.3.2. Dissolution Study

Eudragit RS is a copolymer of ethyl acrylate, methyl methacrylate and a low content of methacrylic acid ester with quaternary ammonium groups. Not soluble in aqueous media across the entire physiological pH range, Eudragit RS exhibits low permeability with pH-independent swelling. It is reported in the literature that Eudragit RS is used extensively in the preparation of matrix tablets for oral sustained release, in tablet coating and in the microencapsulation of drugs [59].

Since both Cloisite and Eudragit RS are insoluble in water, theophylline is released from the granules via a diffusion process. Release of theophylline follows these steps: (1) penetration of the dissolution medium into the theophylline granules, (2) dissolution of theophylline, and (3) diffusion of dissolved theophylline out of the matrix [60].

Since theophylline, a weakly acidic drug, is unable to complex with Cloisite clays, theophylline release from the nanocomposite matrix is controlled by the ionic interactions between the Cloisite and Eudragit RS. As discussed earlier, the ionic interactions reduce the water permeability and equilibrium moisture content of Eudragit RS. Change in drug release rate is also related to the changes in the local permeability due to the molecular level transformation of Eudragit RS in the presence of the silicate sheets [61]. Eudragit RS chains are ionically bound to the dispersed Cloisite layers through the positively charged quaternary amine group. This ionic interaction restricts polymer chain mobility. 

**Figure 10:**
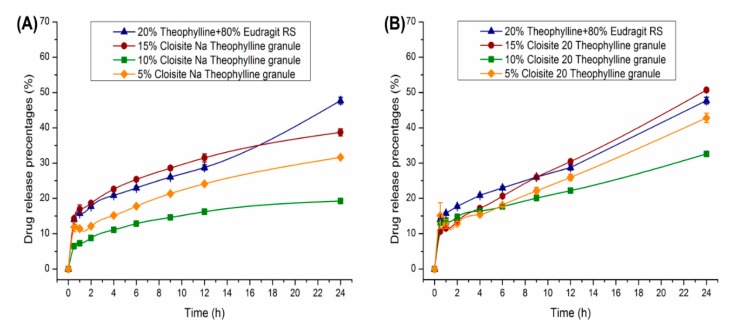
Dissolution profiles of 500 mg theophylline granules 30–35 mesh, 20% theophylline) in 900 mL phosphate buffer pH 6.8 using USP apparatus II at 75 RPM (*n* = 3), (**A**) Cloisite Na nanocomposites of different clay loadings; (**B**) Cloisite 20 nanocomposites of different clay loadings.

Drug release as a function of the clay content in the nanocomposites is plotted in Figure 10. Burst release in the initial 30 min observed for all theophylline granules was due to the release of theophylline located on the surface of the granules. After the initial burst, theophylline release followed a zero-order profile. As shown in Figure 10A, the addition of 5%–10% Cloisite Na in nanocomposites resulted in slower drug release. The percentage theophylline released at 12 h was reduced from 29% (clay-free granules) to 24% (5% Cloisite Na) and 16% (10% Cloisite Na). However, drug release accelerated with further increase of clay content to 15%. The trend in drug release rate as a function of Cloisite Na loading matched well with the DVS results discussed earlier. Equilibrium moisture content decreased with initial increase in Cloisite Na content. Beyond 10%, further increase in clay loading resulted in higher equilibrium moisture content. This increase in equilibrium content at 15% clay loading can be explained by the increasing quantity of nonexfoliated silicate layers that formed tactoids and intercalated structures, as revealed by the TEM and XRPD analyses.

As shown in Figure 10B, the inclusion of Cloisite 20 in the Eudragit RS matrix showed a reduced impact on theophylline release compared to Cloisite Na. After 12 h, the percentage of theophylline released was 29%, 26%, 22% and 30%, for formulations based on clay–polymer nanocomposites containing 0%, 5%, 10%, and 15% Cloisite 20, respectively.

It is concluded that the clay type has a critical influence on drug release profiles. As shown in Figure 10, compared with Cloisite 20, the Cloisite Na is more effective in hindering the release of theophylline. For Cloisite 20, the organic modification hindered its interaction with Eudragit RS.

In summary, the presence of Cloisite nanoclay in the Eudragit RS matrix significantly impacted theophylline release profiles. Clay loading and clay type are the most important factors that would impacts the drug release behaviors. 

**Figure 11:**
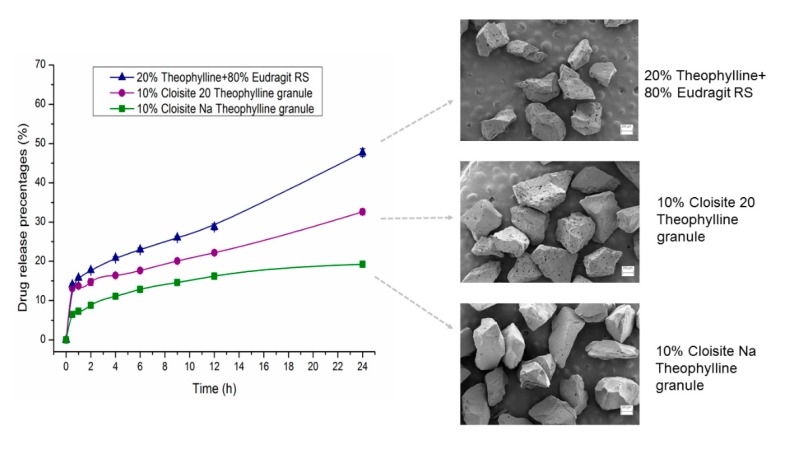
Dissolution profiles of 500 mg theophylline granules (30–35 mesh, 20% theophylline) in 900 mL phosphate buffer pH 6.8 using USP apparatus II at 75 RPM (*n* = 3) and the SEM images of remaining nanocomposites collected at the end of dissolution testing.

As shown in Figure 11, dissolution samples showed different surface morphology after 24 h of dissolution testing. After 24 h of dissolution testing, the surface pore size of the Cloisite 20 samples and Eudragit RS sample is larger than that of the Cloisite Na samples. Differences in porosity are attributed to the different release rates. DSC and XRPD data indicated the Cloisite Na and Cloisite 20 samples had similar crystallinity. We hypothesize that the difference in the porosity was attributed to the difference in the size of the drug crystals dispersed in the extruded granules, since Cloisite Na and Cloisite 20 nanocomposites are water insoluble and the drug particles on the matrix surface are dissolved and released first. Upon exhaustion of the drug on the surface, the depletion zone will then increase progressively as the solid drug front recedes into the matrix while the larger pore size will facilitate the drug release from the matrix. Other important factors that will impact the drug release behaviors are the tortuosity, the molecular level interaction of polymer matrix with silicate layers and the interaction between the API molecule and the nanoclay. There are numerous mechanisms that may be involved in the interaction between clay and organic molecules. The predominant mechanism depends on largely on the type of clay, the functional groups of the polymer and the physical chemical properties of the API [62,63]. 

The current theophylline formulation is by no mean optimized. Further studies are being carried out to identify formulation and process variables to achieve more desired drug release profiles. These variables include granule particle size, drug loading, Eudragit types (RS vs. RL), and incorporation of pore forming agent.

## 4. Conclusions

The study has demonstrated that both Cloisite Na and Cloisite 20 could be exfoliated in Eudragit RS through hot melt extrusion. The XRPD and TEM analyses of the nanocomposites have shown that under the same processing conditions, the nanocomposites’ structures depend on the clay loading and clay structure. When the clay content increases from 5% to 15% by weight, the nanocomposites structures switch from a fully exfoliated state to intercalated structures or partial exfoliation with stacked clay layers. FT-IR results indicated that Cloisite Na and Cloisite 20 layers show different interaction strength with the polymer network, which create a compacted complex structure. DVS data showed that the Cloisite Na nanocomposite is more effective in inhibiting the water absorption than the Cloisite 20 nanocomposite. ssNMR data showed the quaternary ammonium groups of Eudragit RS engaged in the interfacial ionic interaction with the surface negative charged Cloisite clay sheet. Due to the lower cation exchange capacity of Cloisite 20, Eudragit RS has stronger interaction with Cloisite Na. The hygroscopicity difference between Cloisite Na and Cloisite 20 nanocomposites could be attributed to the variation of the interaction between the clay sheet and polymer. The nanocomposites show a high drug encapsulation efficiency, and theophylline exists in a crystal state in the matrix. The addition of nanoclay in the formulation could robustly adjust drug release profiles and the clay concentration and the clay type are the most important factors that impact the drug release behaviors, as they affect the crossing-linking density of the nanocomposites.

## Figures and Tables

**Table 1:** Composition, extrusion torque, and images of extrudates.

Extrusion Time	Formula #	Clay		Polymer	Drug	Average Torque (G.m)	Extrudates Images
		Cloisite Na	Cloisite 20	Eudragit RS	Theophylline		
1st extrusion to prepare clay-polymer nanocomposites	1	-	-	100%	-	883	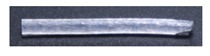
	2	5%	-	95%		841	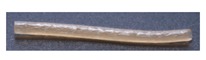
	3	-	5%	95%		751	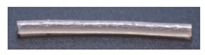
	4	10%	-	90%		984	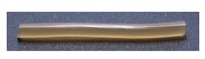
	5	-	10%	90%		761	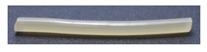
	6	15%	-	85%		1156	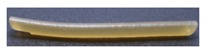
	7	-	15%	85%		887	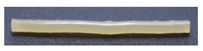
2nd extrusion to incorporate theophylline	1-1	-	-	80%	20%	477	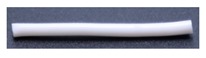
	2-1	4%	-	76%		781	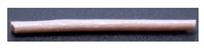
	3-1	-	4%	76%		663	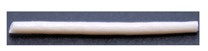
	4-1	8%	-	72%		1202	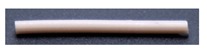
	5-1	-	8%	72%		734	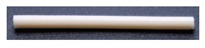
	6-1	12%	-	68%		1436	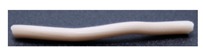
	7-1	-	12%	68%		873	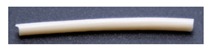

Screw speed: 100 rpm; feed rate: 300 g/h.

**Table 2:** Band assignment for Cloisite Na, Cloisite 20A, and Eudragit RS.

Components	IR Absorption Band (cm^−1^)	Band Assignment
Cloisite Na	1636	δ (O-H) for adsorbed H_2_O
	1078	γ (Si-O) (out-of-plane)
	1007	γ (Si-O) (in-of-plane)
	919	δ (Al-Al-OH)
Cloisite 20	1645	δ (O-H) for adsorbed H_2_O
	1467	δ (C-H) of Aliphatic
	1080	γ (Si-O) (out-of-plane)
	1035	γ (Si-O) (in-of-plane)
	1027	γ (Si-O) (in-of-plane)
	919	δ (Al-Al-OH)
Eudragit RS	1728	δ(C=O) for ester group
	1448	δ(C-H) of alkyl chains
	1386	δ(C-H) of alkyl chains
	1238	γ (O=C-O) for ester group
	1147	γ (O=C-O) for ester group

δ = Bending vibration; γ = Stretching vibration.

## Supplementary Materials

The following are available online at https://www.mdpi.com/1999-4923/12/1/51/s1, Figure S1: FTIR profiles of Cloisite 20 extrudates. (A) From bottom to top, (a) 5% Cloisite 20%–95% Eudragit RS physical mixture; (b) Cloisite 20 extrudates at 5% clay loading; (c) Cloisite 20 extrudates at 10% clay loading; (d) Cloisite 20 extrudates at 15% clay loading. (B) From bottom to top, (a) 5% Cloisite 20%–95% Eudragit RS physical mixture; (b) Cloisite 20 nanocomposite at 5% clay loading; (c) Cloisite 20 nanocomposite at 10% clay loading; (d) Cloisite 20 nanocomposite at 15% clay loading, Figure S2: DVS profiles comparison of Cloisite Na, Eudragit RS and Cloisite 20.
