# Supplementary material for: Clay-Polymer Nanocomposites Prepared by Reactive Melt Extrusion for Sustained Drug Release
Source: Pharmaceutics. 2020 Jan 7;12(1):51. doi: 10.3390/pharmaceutics12010051 (PMC7022276; doi:10.3390/pharmaceutics12010051)
Supplement: Supplementary file 1 [file pharmaceutics-12-00051-s001.pdf]

pharmaceutics

MDPI

Supplementary Materials: Clay-Polymer Nanocomposites Prepared by Reactive Melt Extrusion for Sustained Drug Release

Xu Liu, Xingyu Lu, Yongchao Su, Eucharist Kun and Feng Zhang

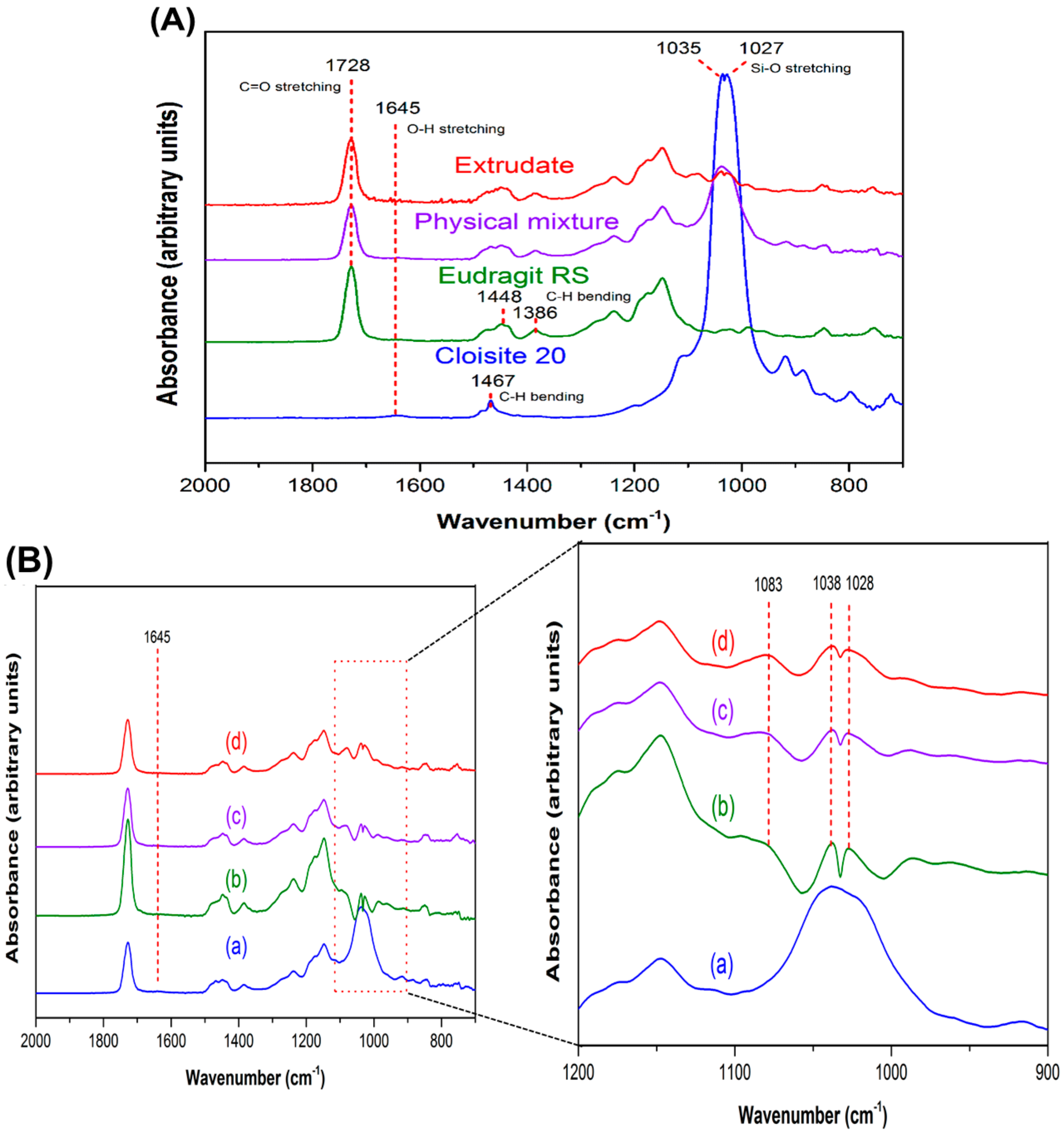

Figure S1. FTIR profiles of Cloisite 20 extrudates. (**A**) From bottom to top, (a) 5% Cloisite 20-95% Eudragit RS physical mixture; (b) Cloisite 20 extrudates at 5% clay loading; (c) Cloisite 20 extrudates at 10% clay loading; (d) Cloisite 20 extrudates at 15% clay loading. (**B**) From bottom to top, (a) 5% Cloisite 20-95% Eudragit RS physical mixture; (b) Cloisite 20 nanocomposite at 5% clay loading; (c) Cloisite 20 nanocomposite at 10% clay loading; (d) Cloisite 20 nanocomposite at 15% clay loading.

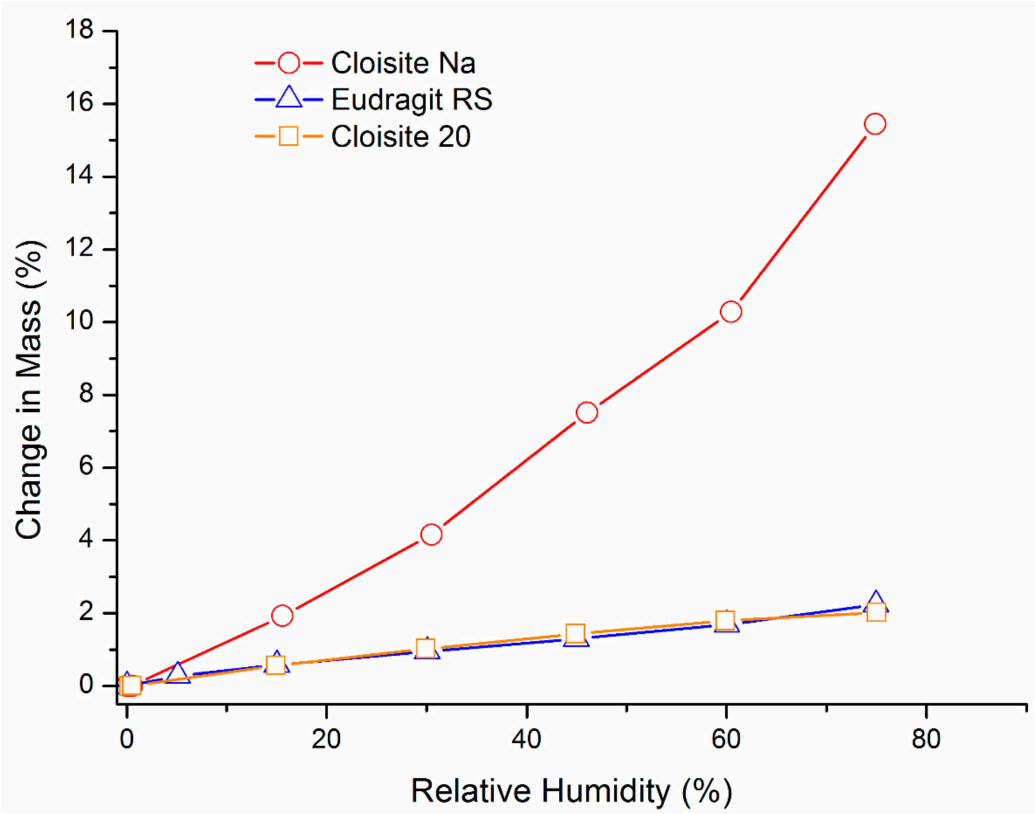

Figure S2. DVS profiles comparison of Cloisite Na, Eudragit RS and Cloisite 20.
